# Serious leisure and successful aging among elderly air volleyball players: examining the mediating role of social support and flow experience

**DOI:** 10.3389/fpsyg.2024.1403373

**Published:** 2024-08-12

**Authors:** Jingzhong Wang, Haibo Tian

**Affiliations:** ^1^Department of Public Basics Teaching, Zhejiang Industry Polytechnic College, Shaoxing, China; ^2^Department of Physical Education, School of Teacher Education, Shaoxing University, Shaoxing, China

**Keywords:** serious leisure, successful aging, air volleyball players, social support, flow experience

## Abstract

**Introduction:**

Previous literature has demonstrated that engagement in serious leisure (SL) is associated with subjective well-being among older adults, while the relationship between successful aging (SA) and SL remains unexplored. This study aims to investigated the association between SL, social support (SS), flow experience (FE), and SA.

**Methods:**

A total of 435 older adults participating in air volleyball events were included in this study.

**Results:**

The findings revealed that: (i) SL directly and positively influences on SS, FE, and SA; (ii) SS is positively related to FE, and FE is positively associated with older adults’SA; (iii) Both SS and FE fully mediate the relationship between SL and SA, with SS partially explaining this mediation through FE.

**Discussion:**

This study builds upon prior research in this field and highlights the significance of SL for the SA among older adults. Future studies should further explore the underlying mechanisms linking serious sport experiences to successful elderly life.

## 1 Introduction

According to the findings of the 7th Chinese National Census (CNC), the proportion of individuals aged 60 years and above reached 18.7% (i.e., 264.02 million) by the end of 2020, representing a 5.44% increase compared to the 6th CNC (2010) data. The global phenomenon of population aging will pose numerous challenges to societies worldwide, including a significant rise in governmental social burdens, a decline in labor supply, and a weakening of familial elderly care function (Morgan and Kunkel, 2007). Recent studies suggest that promoting physical activity can effectively address age-related degradation (Billot et al., 2020). Adapted from traditional volleyball, air volleyball has emerged as a prevalent leisure activity among Chinese adults, offering various physical and psychological health benefits (He, 2009). Therefore, it is imperative to explore how participation in leisure sports contributes to successful aging (SA).

Previous studies have established a strong association between serious engagement in leisure sports activities and the aging process (Heo et al., 2012; Sala et al., 2019). Recent qualitative research has identified four themes through which serious sport participation can promote active aging, namely positive emotions, body improvement, social interactions, and optimistic life attitudes (Zhou et al., 2023). Another study has highlighted the important link between serious leisure (SL) and SA by examining the benefits and costs of senior fashion modeling (Lee et al., 2019). Furthermore, existing literature has explored the relationship between SL and SA by investigating the mediating role of subjective well-being. For instance, subjective well-being was found to mediate the effect of SL on active aging among Chinese Tai Chi participants (Shang and Qiu, 2023), while family structure moderated the relationship between serious leisure involvement and SA for participants in Chinese Seniors’ Universities (Wu et al., 2020). Additionally, Stebbins suggested that psychological flow and social support (SS) were significant components of participants’ leisure career experiences (Stebbins, 2007). In other words, previous studies has provided specific evidence to support the influence of SL on SA, and certain significant variables such as flow experience (FE) and SS may indirectly impact the relationship between SL and SA. Gas volleyball is popular among the elderly due to its lower difficulty, ease of learning, moderate intensity, and strong appeal. It can assist the elderly in maintaining an active lifestyle, finding enjoyment and a sense of accomplishment in sports, as well as enhancing social skills and overall quality of life. ‌However, current research has made limited efforts to investigate the role of SL on SA with respect to air volleyball or using flow experience (FE) and SS as mediating variables.

Therefore, this study aims to provide a novel perspective for comprehending the impact of SL on SA among senior air volleyball players. This research primarily investigates the following aspects: Firstly, does SL has a direct and positive influence on SS, FE, and SA? Secondly, do SS and FE mediate the relationship between SL and SA? Hence, this paper will assess the associations among SL, SS, FE, and SA in elder air volleyball players by proposing a hypothesis model.

## 2 Literature review

### 2.1 Serious leisure and successful aging

Previous studies have introduced the concept of ‘serious leisure’ to capture how individuals assess the significance of leisure activities in their daily lives. This term refers to the deliberate pursuit of amateur, hobbyist, or volunteer activities that are sufficiently substantial, interesting, and fulfilling for participants to develop a (leisure) career by acquiring and expressing a combination of specialized skills, knowledge, and experience (Stebbins, 1982; Elkington and Stebbins, 2014). SL is further characterized by six distinct characteristics including (a) need to persevere, (b) career development, (c) significant personal effort, (d) various durable benefits, (e) unique ethos, and (f) strong sense of identity. These characteristics have been identified across various leisure activities such as marathon running (Ronkainen et al., 2017; Qiu et al., 2020), dance (Schneider and McCoy, 2018; Schupp, 2019), pickleball (Heo et al., 2018), gardening (Cheng et al., 2017), and rugby ball sports (Cheng et al., 2016a,b).

SA mainly reflects a later life of sustained health and vitality where older people is helpful to society rather than a lifetime of dependency and ill health (Achenbaum, 2001). While there has been ongoing debate regarding the definition of SA, an increasing number of studies have adopted a subjective perspective rather than an objective approach (Godbey et al., 2005; Estebsari et al., 2020). A recent study defined it as achieving a relatively high level of physical, psychological, social, and spiritual well-being through adaptation (Lee et al., 2016). A systematic review concluded that most studies aimed to measure successful aging using a multidimensional framework which offers greater opportunities compared to unidimensional approaches for identifying areas requiring improvement (Cosco et al., 2014).

Existing literature has primarily focused on defining SA and exploring its determinants (Ng et al., 2009; Estebsari et al., 2020). However, limited attention has been given to investigating the influential factors of SA in the context of leisure activities. Stebbins concluded that serious participants derive numerous enduring benefits such as self-actualization, enhanced self-image, and social interaction from overcoming challenges and exerting significant personal efforts (Stebbins, 1982, 2007; Elkington and Stebbins, 2014). Qualitative researches have suggested that engaging in serious leisure activities can enhance well-being among older adults and promote a healthy lifestyle (Kim et al., 2014; Son et al., 2021). Similarly, Zhou et al. (2023) conducted a thematic analysis which revealed that older Chinese adults experienced various long-lasting outcomes including physical improvements, positive life attitudes, emotional well-being, and increased social interactions through their involvement in serious leisure sports.

### 2.2 Social support and its’ mediating effect

The concept of SS refers to the process of interaction within relationships that enhances coping mechanisms, self-esteem, a sense of belonging, and competence through actual or perceived exchanges of physical or psycho-social resources (Cohen et al., 2000). SS is typically provided by individuals in one’s social network and is often associated with the frequency and extent of communication with friends, family members, and significant others (O'Reilly, 1988; Due et al., 1999). Functionally, SS can be categorized into four types: emotional support, informational support, appraisal support, and instrumental support (House et al., 1988; Berkman et al., 2000). Zimet et al. developed a three-dimensional scale to measure perceived social support which demonstrated good internal reliability and moderate construct validity (Zimet et al., 1988).

The commitment of individuals to a leisure activity is not an independent occurrence, but rather influenced by the social context (Lee, 2020). Serious participants in the social world share similar attitudes, values, beliefs, practices, and goals through various communication channels (Veal, 2017), such as the internet and magazines (Cox and Blake, 2011; Hughes et al., 2016), or club communications (Gillespie et al., 2002). They typically seek SS from friends, family, and significant others while engaging in their leisure pursuits (Lee et al., 2017). For instance, a qualitative study revealed that older participants received greater support for enhancing their social interactions like communication and group maintenance through involvement in SL sport activities (Zhou et al., 2023).

Social networks and SS exert a direct influence on the well-being and health of adults (Hombrados-Mendieta et al., 2019). A 20-year longitudinal study revealed that robust SS serves as one of the most influential predictors for SA (Bosnes et al., 2019). Previous research has confirmed that actively engaging in leisure activities significantly contributes to acquiring SS from friends (Lee et al., 2017). The withdrawal of older adults from key domains of social life leads to a decline in interpersonal relationships, resulting in feelings of loneliness. Consequently, older adults are more inclined to enhance their quality of life through active participation in leisure sports activities, partially facilitated by accessing SS from family members, friends, and significant others.

### 2.3 Flow experience and its’ mediating effect

Flow experience, as a key research area in the field of positive psychology, was developed to elucidate why individuals engage in activities without any economic or material incentives (Csikszentmihalyi, 1975). It is defined as an enjoyable and intrinsically rewarding psychological state wherein individuals experience complete absorption in an activity, exclusion of irrelevant thoughts, and a sense of seamless integration even amidst challenging situations (Csikszentmihalyi, 2022). FE commonly revolves around a nine-dimensional framework (Tse et al., 2021), with six dimensions describing its phenomenology (e.g., clear goals, balance between challenge and skill, and unambiguous feedback) and three dimensions delineating the preconditions for experiencing flow (e.g., sense of control, transformation of time perception, and autotelic experiences). In the realm of sports research, most studies have focused on interventions aimed at enhancing FE (Aherne et al., 2011; Koehn et al., 2014), antecedents that contribute to the occurrence of flow states (Swann et al., 2012; Koehn et al., 2018), as well as the impact of flow on performance outcomes (Ferreira et al., 2008).

FE may manifest in SL activities due to their provision of significant challenges or benefits, such as self-enrichment, self-expression, and self-actualization (Mannell, 1993; Elkington and Stebbins, 2014). The results of a recent study revealed a positive correlation between the daily engagement in SL activities and FE among older adults participating in various programs, such as intergenerational activities, volunteer opportunities, and support groups (Heo et al., 2010). Another study has also confirmed that a higher level of leisure involvement is associated with a stronger FE among mountain hikers in Taiwan (Cheng et al., 2016b). Utilizing structural equation modeling, Frash and Blose discovered that the enduring benefits (i.e., a primary facet of SL) exerted a positive influence on the FE experienced by serious motorcycle tourists (Frash and Blose, 2019).

Previous research has demonstrated that the FE in individuals’ leisure activities can significantly contribute to their overall life satisfaction and subjective well-being (Tian et al., 2022a,c). A body of quantitative and qualitative studies has provided evidence suggesting that engaging in flow-inducing exercise can lead to positive improvements in various outcome variables, including physical fitness (Karageorghis et al., 2000), sense of coherence (Iida and Oguma, 2014), confidence (Swann et al., 2019), and overall well-being (Iida and Oguma, 2013). Collectively, these findings tentatively suggest a potential association between FE and a range of desirable benefits related to SA. In other words, it is plausible that SL pursuits may facilitate high levels of SA among elderly individuals, with FE indirectly promoting this relationship.

### 2.4 Purpose of this study

**Figure 1:**
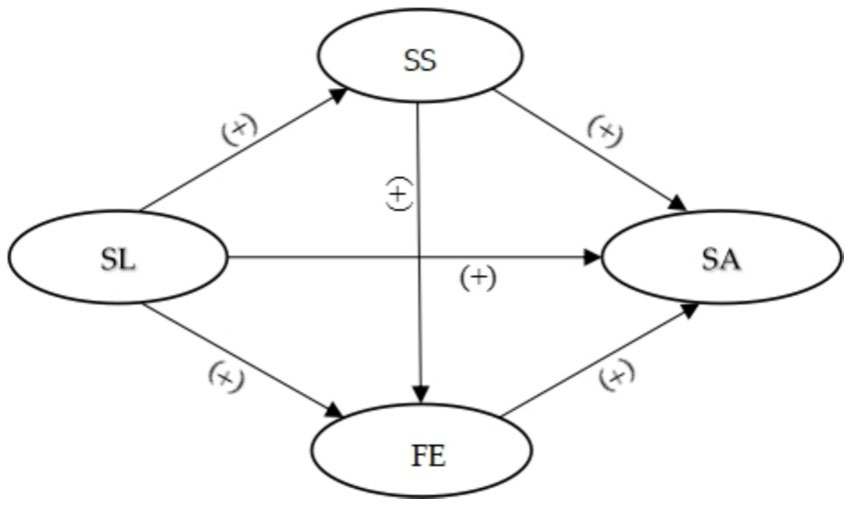
Conceptual model of research hypotheses.

The current study was designed to quantitatively estimate the association between serious leisure and successful aging among older Chinese adults. Therefore, this study aims to explore the relationship between SL, SS, FE, and SA among older Chinese adults who were members of air volleyball clubs. Based on the preceding discussion, the research hypotheses in this study are as follows (See Figure 1):

*H1:* SL is positively associated with SS.

*H2:* SL has a positive influence on the FE.

*H3:* SL directly contributes to SA.

*H4:* SS significantly contributes to SA.

*H5:* FE has a direct impact on SA.

*H6:* There is a positive association between SS and FE.

*H7:* SL indirectly influences SA by enhancing SS.

*H8:* SL indirectly impacts SA through its facilitation of FE.

## 3 Methods

### 3.1 Participants

Based on the experience of previous studies (Heo et al., 2018; Tian et al., 2023), this study included elder adults aged 60 years old and above. A total of 453 paper questionnaires were collected, but 18 of the data were excluded from this study based on Qiu et al.’s (2020)operational definition of serious leisure, as they did not meet the criterion of having insisted to play air volleyball for 6 months. Ultimately, data from 435 Chinese participants (*M* = 70.154; SD = 5.517) were used to analyze and examine all research hypotheses in this srudy. The demographic characteristics of the participants are presented in Table 1. A majority of the participants were male (243 or 55.9%). More than half of the participants belonged to the age group of 66 years and above (264 or 60.7%). The majority of respondents were married (413 or 95.6%), while a smaller proportion were unmarried (3 or 0.7%) or divorced/widowed (19 or 4.4%). Over half of the respondents had attained a college or university education level (240 or 55.2%), with a smaller proportion having a postgraduate education level (8 or 1.8%). Approximately, 87.4% of participants reported having two or more siblings, whereas only a minority reported major diseases (90.1%). Overall, except for an imbalance in marital status, our research sample can be considered representative.

**Table 1:** The information of demographic characteristics (*n* = 435).

Characteristics	Frequency (*n*)	Percentage (%)
Gender		
Male	243	55.9
Female	192	44.1
Age		
61–65	171	39.3
66–70	97	22.3
71–75	80	18.4
76 and above	87	20.0
Marital status		
Unmarried	3	0.7
Married	413	95.6
Divorced or widowed	19	4.4
Education		
High school or below	187	43.0
College or university	240	55.2
Postgraduate	8	1.8
Number of siblings		
1 and below 2–3	55 265	12.6 60.9
4 and above	115	26.4
Absence of major diseases	392	90.1

### 3.2 Measurement

The Serious Leisure Inventory and Scale (SLIM), developed by Gould et al. (2011), was used to access the level of SL for elder air volleyball players. SLIM comprises six dimensions and includes a total of 18 items, encompassing perseverance (1 item), personal effort (1 item), unique ethos (1 item), strong identity (1 item), career (2 items), and durable benefits (12 items). For example, a statement related to perseverance is exemplified as ‘I demonstrate persistence in overcoming difficulties in air volleyball.’ The scoring for these items was conducted on a 5-point Likert scale ranging from 1 (strongly disagree) to 5 (strongly agree). In this study, the overall internal consistency reliability coefficient of SLIM was found to be 0.94, which aligns with the value reported by Gould et al. (2011) at 0.97.

The Multidimensional Scale of Perceived Social Support (MSPSS), developed by Zimet et al. (1988), was utilized to evaluate the respondent’s perception of SS. The MSPSS consisted of 12 items, which were further classified into three dimensions: significant other (4 items), friends (4 items), and family (4 items). For the significant other dimension, participants were requested to indicate their level of agreement with the statement ‘I have companions with whom I can share my joys and sorrows.’ Ratings on the MSPSS were collected using a 7-point Likert scale ranging from ‘strongly disagree’ (1) to ‘strongly agree’ (7). The Cronbach’s α coefficients for the SS factors ranged from 0.80 to 0.93. Confirmatory factor analysis (CFA) results yielded χ^2^ = 232.15, df = 51, RMSEA = 0.06, GFI = 0.90, CFI = 0.94, TLI = 092.

The Flow Short Scale (FSS), adapted from previous studies (Rheinberg et al., 2003; Engeser and Rheinberg, 2008), was employed to measure the flow state of air volleyballs players. FSS consists of 10 items, including absorption by activity (4 items) and fluency of performance (6 items). The items are rated on a 7-point Likert scale where ‘1’ represents ‘not at all’ and ‘7’ represents ‘very much’. The statement for FE was as follow: ‘I feel just the right amount of challenge’. Higher total scores on the FSS indicate greater level of flow experience. The Cronbach’s α coefficients for FE dimensions are 0.84 and 0.94, respectively. CFA results yielded satisfactory results: χ^2^ = 86.99, df = 17, RMSEA = 0.09, GFI = 0.94, CFI = 0.96, TLI = 0.94.

The Successful Aging Scale-Chinese version (SAS-C), developed by Lee et al. (2016) specifically for the context of Taiwan province, was employed to measure elderly adults’ subjective perception of physical, psychological, social, and spiritual well-being quality. The SAS-C comprised 20 items across four dimensions: physical well-being (5 items), mental well-being (5 items), social well-being (5 items), and spiritual well-being (5 items). The statement for physical well-being, for instance, was ‘I can take care of my own daily life’. The items were assessed using a 5-point Likert scale ranging from ‘strongly disagree’ (score 1) to ‘strongly agree’ (score 5). In this study, the SAS-C demonstrated good reliability coefficients ranging from 0.79 to 0.88. CFA results yielded the following results: χ^2^ = 159.96, df = 34, RMSEA = 0.10, GFI = 0.91, CFI = 0.93, TLI = 0.95.

Demographic variables. Building upon the previous research findings (Şimşek et al., 2021; Tian et al., 2023), a 6-item demographic variables was developed by the researchers, encompassing gender, age, marital status, education level, number of siblings, and absence of major diseases.

### 3.3 Procedures and data analysis

The data for this study were collected during the Air Volleyball Event of the 8th Zhengjiang Senior Sport Game (AVEZSSG) held from October 9 to 19 in Shaoxing and Jinhua, two prominent cities in Zhejiang province. This event has gained increasing attention and popularity among the elderly population since 1988, ultimately attracting a total of 36 representative teams from across the province. Considering the event’s magnitude, a random sampling technique was employed in this study, with questionnaires distributed among air volleyball players or enthusiasts near the designated rest area at intervals of every third person. Prior to administering the questionnaire, respondents were requested to provide informed consent by signing a consent form.

The data in this study were analyzed using SPSS 23.0 and AMOS 24.0 software packages. Descriptive analysis was conducted to assess the frequency of demographic variables, as well as the mean and standard deviations. Cronbach’s alpha (CA) was employed to evaluate the reliability of all variables in this study. Pearson’s correlation coefficient was utilized to examine the correlation among all variables. CFA in AMOS was performed to test the construct validity of all sub-scales. Following Hayes’s (2017) suggestions, hypotheses 1 to hypothesis 8 were carried out using the boostrap method of 5,000 samples using PROCESS Macro Analysis v4.0 which was installed on SPSS 23.0.

## 4 Results

### 4.1 Descriptive statistics and correlation analysis

The results of descriptive statistics and correlation analysis for each construct are presented in Table 2. SS exhibited a higher mean score (*M* = 5.558, SD = 0.599), followed by FE (*M* = 5.481, SD = 0.509). In contrast, SL (*M* = 4.015, SD = 0.592) and SA (*M* = 4.161, SD = 0.275) demonstrated lower mean scores. These findings strongly indicate that the elderly population displayed a significant level of SS, FE, and SA due to their active participation in gas volleyball activities. Furthermore, it was observed that both SS and FE increased with an increase in SL (*r* = 0.366–0.368; *p* < 0.01), while SA showed positive correlations with SL as well as SS and FE (*r* = 0.366–0.860; *p* < 0.01).

**Table 2:** Descriptive statistics and correlation of variables (*n* = 435).

Constructs	M ± SD	SL	SS	FE	SA
SL	4.015 ± 0.592	--			
SS	5.558 ± 0.599	0.366**	--		
FE	5.481 ± 0.509	0.368**	0.647**	--	
SA	4.161 ± 0.275	0.420**	0.774**	0.860**	--

(1)** *p*<0.01; (2) SL = serious leisure; SS = social support; FE = flow experience; SA = successful aging.

### 4.2 Research hypothesis testing

**Figure 2:**
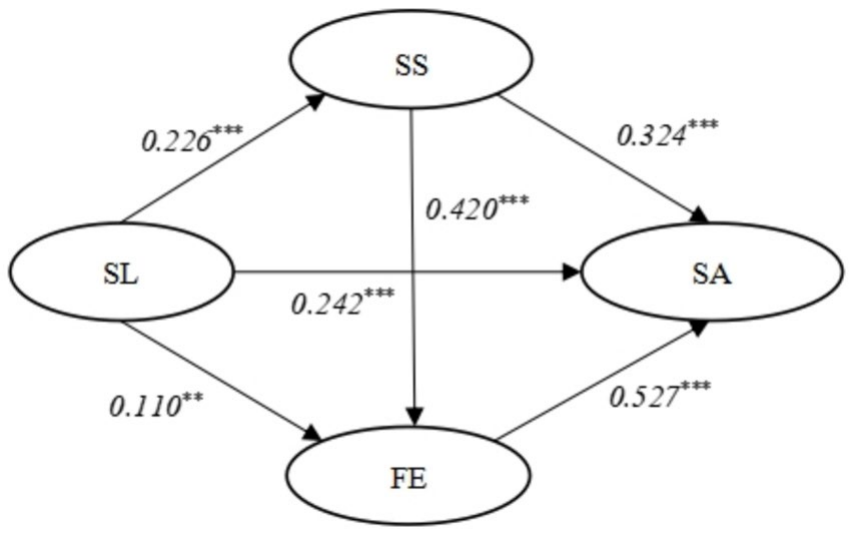
Standardized parameter estimate of conceptual model. **p < 0.01;***p < 0.001.

According to Figure 1, SL was utilized as an independent variable, while SS and FE were employed as mediating variables. SA served as the dependent variable, with demographic variables included as control variables. As depicted in Table 3 and Figure 2, the results indicate a significant positive relationship between SL (*β* = 0.226, *p* < 0.001), SS (*β* = 0.324, *p* < 0.001), FE (*β* = 0.527, *p* < 0.001) and SA. Therefore, these findings provide support for hypotheses H3, H4, and H5. Moreover, it is noteworthy that both SL (*β* = 0.110, *p* < 0.01) and SS (*β* = 0.420, *p* < 0.001) exert a significant positive impact on FE, while SL (*β* = 0.226, *p* < 0.001) also demonstrates a favorable influence on SS. Consequently, these empirical findings lend support to hypotheses H1, H2, and H6.

**Table 3:** Standardization path coefficient and hypothesis testing results (*n* = 435).

Hypothesis	*β*	SE	*t*	*p*	Decision
H1: SL → SS	0.226	0.042	5.460	<0.001	Support
H2: SL → FE	0.110	0.031	3.094	<0.01	Support
H3: SL → SA	0.242	0.016	6.893	<0.001	Support
H4: SS → SA	0.324	0.012	12.453	<0.001	Support
H5: FE → SA	0.527	0.015	18.913	<0.001	Support
H6: SS → FE	0.420	0.034	10.416	<0.001	Support

SL, serious leisure; SS, social support; FE, flow experience; SA, successful aging.

The mediating effects were assessed, and the 95% confidence intervals were computed as presented in Table 4. The study findings provide evidence that both SS and FE significantly mediate the relationship between SL and SA, as indicated by the exclusion of zero from all confidence intervals. Therefore, based on the data analysis results, H7 (a*b = 0.073, 95%CI = −0.271 ~ −0.659) and H8 (a*b = 0.058, 95%CI = 0.019 ~ 0.096) are supported. Furthermore, considering the direct effect result (95%CI = −0.047 ~ −0.009), it can be concluded that SS and FE fully mediate the impact of SL on SA.

**Table 4:** Bootstrap analysis on the testing mediation effect (*n* = 435).

Hypothesis test	Effect value	Bootstrap SE	95% CI	
			Lower	Upper
Total effect	0.209	0.016	0.081	0.145
Direct effect	0.028	0.010	−0.047	−0.009
Indirect effect	0.181	0.074	0.598	0.891
SL → SS → SA	0.073	0.015	−0.105	−0.045
SL → FE → SA	0.058	0.019	0.019	0.096
SL → SS → FE → SA	0.050	0.011	0.029	0.073

(1) Using bootstrap method, 5,000 bootstrap re-sampling; (2) SL, serious leisure; SS, social support; FE, flow experience; SA, successful aging.

## 5 Discussion

The aim of this study was to examine the contributions of SL, SS, and FE to SA in the context of air volleyball among older adults. Results from the Process v4.0 procedure provided support for all eight hypotheses posited in this study. A significant finding revealed that an increase in SL significantly enhanced SA. These findings align with previous research indicating a positive association between leisure involvement, recreation specialization, and SA (Wu et al., 2020; Tian et al., 2022a,b). Extending to existing studies (Lee et al., 2019; Zhou et al., 2023), our quantitative research method further confirms the role of SL in promoting SA. Silverstein and Parker (Silverstein and Parker, 2022) recommended meaningful engagement in leisure activities as an adaptive strategy employed by older adults to compensate for physical and social deficits later in life. When older adults actively participate in their chosen leisure pursuits, they can experience tangible benefits such as self-renewal, a sense of belongingness, and improved self-image (Elkington and Stebbins, 2014).

Our study findings demonstrate the mediating role of SS in the relationship between SL engagement and SA. These results provide further clarification to Kim et al.’s (Kim et al., 2014) research, which highlighted that individuals who actively participate in specific physical activities can experience various health benefits, including the establishment of social support networks that contribute to SA. Additionally, Zhang et al. emphasized the significant mediating effect of SS on the association between leisure activities and mental health among older adults (Zhang et al., 2021). Consistent with prior literature, engaging in leisure seriously has a positive association with gaining social support from friends (Lee et al., 2017), and gains various benefits related to successful aging, such as interpersonal relationship (Brown et al., 2008), social interaction (Siegenthaler and O'Dell, 2003). Moreover, the existing literature has not yet to provide a clear understanding of how SL contributes to SA, particularly in terms of the mediating role played by SS. Building upon previous studies (Wu et al., 2020; Tian et al., 2022a,b, this study confirms the mediating effect of SS on the relationship between SL and SA.

In line with previous research (Heo et al., 2010; Frash and Blose, 2019), the level of FE among older air volleyball players increased in accordance with their degree of engagement in SL activities. Flow is characterized as an “autoletic” experience, or the sensation that arises from engaging in intrinsically rewarding activities (Stebbins, 2007). Conceptualized as “situational involvement” within the field of leisure studies (McIntyre, 2003), flow was found to be enhanced when individuals experienced various long-term benefits such as self-enrichment, self-gratification, and self-actualization (Cheng et al., 2016b). Furthermore, this study supports existing literature findings (Heo et al., 2010; Lee, 2012) that FE significantly contributes to subjective well-being and SA among elderly individuals. The balance between skill and challenge experienced during air volleyball engagement appears to rejuvenate older adults to some extent. Moreover, this study confirms that FE mediates the relationship between SL and SA through two pathways: indirect effects generated by FE itself and chain mediating effects involving SS and FE. These findings contribute to existing literature (Heo et al., 2010; Lee, 2012; Frash and Blose, 2019) by providing insights into how SL influences SA among elderly individuals participating in sports activities. Specifically, strengthening SS networks and promoting FE can indirectly enhance SA outcomes for older volleyball players.

Compared with traditional aerobic sports such as fast walking and Tai chi, Gas volleyball offers the eldly a unique and enduring leisure experience that promotes their active participation. Analytical findings suggest that SL plays a significant role in predicting SA among elderly air volleyball players. Therefore, it is recommended that air volleyball managers organize regular and diverse volleyball activities to enhance its appeal. This strategic approach can greatly contribute to the attainment of long-lasting benefits associated with SA, such as a healthy lifestyle and self-actualization. Furthermore, SS acts as a mediator between SL and SA, highlighting the importance for managers to establish online or offline volleyball community groups where participants can exchange their leisure experiences based on shared interests, skills, and beliefs. Additionally, this study reveals that FE mediates the relationship between SL and SA among older adults. Consequently, event managers should flexibly adjust event schedules to promote skill development while providing appropriate challenges for seniors.

The present study has identified several limitations and offers suggestions for future research. Firstly, the investigation focused on players engaged in air volleyball activity to explore the relationship between SL, SS, FE, and SA. However, it is important to note that these findings may not be generalizable to participants involved in different types of leisure sport activities. These hypotheses should be evaluated in other types of activities for future research. Additionally, recent studies have suggested that variables such as psychological commitment and loneliness significantly influence life satisfaction and SA (Cheng et al., 2016b; Tian et al., 2022a,b. Therefore, this study proposes that future research should incorporate these variables to further elucidate the relationship between SL, psychological commitment, loneliness, and SA. Lastly, this study employed a random sampling method to recruit elderly air volleyball players from Zhejiang Province; however, it is important to note that the sample may not be fully representative of all air volleyball players in China. Therefore, future research should aim to encompass a broader geographic area and include other types of volleyball events.

## 6 Conclusion

This study aimed to investigate the role of SL on SA, with FE and SS as mediators among elderly air volleyball players. Our findings contribute to a better understanding of the link between SL and SA by demonstrating that it has a positive impact on SS, FE, and SA. Furthermore, our results suggest that SS and FE mediate the relationship between SS and SA. These findings shed light on how SL influences the process of SA among older adults in China, providing both theoretical insights and practical implications for enhancing their quality of life.

## Data Availability

The original contributions presented in the study are included in the article/supplementary material, further inquiries can be directed to the corresponding author.
